# Relationship of sex differences in cortical thickness and memory among cognitively healthy subjects and individuals with mild cognitive impairment and Alzheimer disease

**DOI:** 10.1186/s13195-022-00973-1

**Published:** 2022-02-22

**Authors:** Filippo Cieri, Xiaowei Zhuang, Dietmar Cordes, Nikki Kaplan, Jeffery Cummings, Jessica Caldwell, for the Alzheimer’s Disease Neuroimaging Initiative (ADNI)

**Affiliations:** 1 Cleveland Clinic Lou Ruvo Center for Brain Health, Las Vegas, NV USA; 2 Interdisciplinary Neuroscience Program, University of Nevada Las Vegas (UNLV), Las Vegas, NV USA; 3 University of Colorado Boulder, Boulder, CO USA; 4 Chambers-Grundy Center for Transformative Neuroscience, Department of Brain Health, School of Integrated Health Sciences, University of Nevada Las Vegas (UNLV), Las Vegas, NV USA

**Keywords:** Alzheimer Disease Dementia, Mild cognitive impairment, Sex, Magnetic resonance imaging, Cortical thickness, Memory, RAVLT, Machine learning

## Abstract

**Background:**

An aging society has increased rates of late onset Alzheimer disease dementia (ADD), the most common form of age-related dementia. This neurodegenerative disease disproportionately affects women.

**Methods:**

We use data from the Alzheimer’s Disease Neuroimaging Initiative (ADNI) to examine sex differences in cortical thickness (CT) and memory performance. Analyses of covariance (ANCOVA) models were used to examine effects of sex and diagnosis (DX) on CT and verbal memory. For regions demonstrating significant interaction effects of sex and DX, we tested whether sex moderated cognition-thickness relationships. We used machine learning as a complementary method to explore multivariate CT differences between women and men.

**Results:**

Women demonstrated greater CT in many brain regions. More specifically, men showed relatively consistent CT declines in all stages, from normal control (NC) to ADD in the bilateral cingulate cortex, bilateral temporal regions, and left precuneus; women had more stable CT in these regions between NC and mild cognitive impairment (MCI) stages, but sharper declines from MCI to ADD. Similarly, for the Rey Auditory Verbal Learning Test (RAVLT), ANCOVA analyses showed that women had significantly better immediate and delayed recall scores than men, at NC and MCI stages, but greater differences, cross-sectionally, from MCI to ADD than men. We found significant sex moderation effects between RAVLT-immediate scores and CT of right isthmus-cingulate for all subjects across DX. Partial correlation analyses revealed that increased CT of right isthmus-cingulate was associated with better verbal learning in women, driven by positron emission tomography defined amyloid positive (Aβ+) subjects. Significant sex-moderation effects in cognition-thickness relationships were further found in the right middle-temporal, left precuneus, and left superior temporal regions in Aβ+ subjects. Using a machine learning approach, we investigated multivariate CT differences between women and men, showing an accuracy in classification of 75% for Aβ+ cognitively NC participants.

**Conclusions:**

Sex differences in memory and CT can play a key role in the different vulnerability and progression of ADD in women compared to men. Machine learning indicates sex differences in CT are most relevant early in the ADD neurodegeneration.

**Supplementary Information:**

The online version contains supplementary material available at 10.1186/s13195-022-00973-1.

## Background

The investigation of sex differences has a long tradition in neuropsychology and cognitive neuroscience. This pursuit is important given different vulnerabilities by sex in incidence, symptomatology, and progression of many neurological and psychiatric diseases. Chief among these is late onset Alzheimer’s disease dementia (ADD) [1–7]. Almost 70% of ADD patients are women [8], but the reasons for these sex disparities remain mostly unknown.

One hypothesis of sex disparities in ADD is that women’s brains are more vulnerable to ADD pathology. This is supported by longitudinal studies showing sex differences in volumetric change over time [9, 10], memory trajectories [11], and tau accumulation rates [12]. At the same time, work by our group and others has shown that cognitively normal women have a verbal memory advantage over men [13, 14] that persists in the presence of brain amyloid [1, 2, 4] and mild to moderate ADD pathological burden such as volume loss and brain hypometabolism [14–16]. It is unclear whether sex differences in brain structure or atrophy over time account for this pattern of women’s advantage followed by accelerated decline in memory between the MCI and the ADD stages.

There are few studies exploring sex differences in cortical thickness (CT) in the field of ADD [17, 18], and our knowledge on the role of the ways sex and CT might interact to inform understanding of cognitive changes in ADD is limited. Two concepts that can contribute to this pursuit are brain and cognitive reserve in aging. Brain reserve is defined as structural brain characteristics that protect, resist, or compensate against expression of pathology. Cognitive reserve (CR) refers to features of an individual such as years of education [19], which might provide means to better adapt and maintain cognitive performance despite early pathological brain changes [20].

Women in contemporary ADD studies often present with fewer years of education than men, suggesting general CR measures do not explain sex differences. From this, the question remains as to whether women have a more specific CR in memory and whether there are measurable neural underpinnings—or brain reserve—related to the memory reserve. Findings by our group have provided mixed results for brain reserve in the form of hippocampal volume [1, 3] and more consistent support for resting state functional connectivity differences [4, 5].

Recent classification approaches provide new ways to explore sex differences in brain atrophy using machine learning algorithms [21]. Statistical learning enables researchers to explore statistical patterns to build predictive systems and generalizable models using various features, for example, classifying individual subjects using surface area [22] or volumetric features [23] derived from structural magnetic resonance imaging (MRI).

The current investigation sought to determine sex differences in CT and memory in NC, MCI, and ADD subjects, in a large sample of men and women from the ADNI database. We hypothesized that CT declines over time in brain regions involved in memory and known to be impacted by ADD would show sex differences and that memory trajectories would differ by sex in a parallel fashion. We expected CT in brain regions implicated in the ADD pathology to relate to memory scores, particularly in women. We used machine learning as a complementary method to explore multivariate CT differences between women and men. We hypothesized that machine learning would differentiate the brains of men and women, with classification accuracies reducing as ADD progresses. We expected our hypotheses to apply best in individuals with confirmed brain amyloid aggregation, defined by amyloid Positron Emission Tomography (PET).

## Materials and methods

### Participants and data collection

A large sample of subjects from the ADNI database (http://www.adni-info.org) were included. Briefly, the ADNI is a multicenter, multi-phase study assessing clinical, imaging, and genetic biomarkers in AD. We assessed all 838 older participants with normal cognition (NC) and subjects with mild cognitive impairment (MCI) or ADD from the ADNI2/GO database, who had available clinical diagnosis, genetic information, verbal memory assessments, 3T structural MRI, and florbetapir amyloid PET imaging at the same visit. ADD subjects with a negative amyloid status were excluded from the study due to the potential presence of pathologies other than ADD. Therefore, we included a final sample of 265 NC subjects, 442 MCI subjects, and 117 ADD subjects (total 824 subjects). NC and MCI subjects were further divided into amyloid negative (NCAβ- (*N*=177) and MCIAβ- (*N*=191)) and amyloid positive (NCAβ+ (*N*=88) and MCIAβ+ (*N*=251)) groups; all ADD subjects had positive amyloid status (ADDAβ+ (*N*=117)).

### Demographics and ApoE genotype

Subjects’ demographics including sex, age, years of education (YOE), handedness, and ApoE genotype were downloaded from the ADNI website. Two categorical variables were created to code individuals’ ApoE-*ε*2 (*ε*2) and *ε*4 carrier status, respectively, with the presence or absence of at least 1 copy of the ApoE-*ε*2 or *ε*4 allele.

### Verbal memory assessment

Rey Auditory Verbal Learning Test (RAVLT) from the same visit as clinical diagnosis was used to assess verbal learning and memory performances. Both total learning score across five learning trials (RAVLT-Immediate) and delayed free recall scores (RAVLT-Delayed) were used.

### Structural MRI processing

Fully processed CT data for all brain regions, at the same visit as clinical diagnosis, were downloaded from ADNI, with methods described in the UCSF FreeSurfer Methods Quality Control document (www.adni.loni.usc.edu). Briefly, subject-specific T1-weighted MRI images at the corresponding visit were preprocessed by the Mayo Clinic, and FreeSurfer (version 5.1 http://surfer.nmr.mgh.harvard.edu/) was then employed to generate a subject-specific anatomical labeling. Cortical thickness measures of 68 cortical regions [24] were finally obtained. Details of these 68 regions are listed in Supplement S1.

### Florbetapir PET image processing

Subjects’ amyloid status were determined from the PET florbetapir images at the same visit as clinical diagnosis. The summarized standardized uptake value ratio (SUVR) normalized to the cerebellum were obtained from the ADNI database and amyloid positivity status was defined as the global SUVR greater than 1.1.

### Statistical analysis

All statistical analysis was conducted in matrix laboratory (MATLAB) 2018b (https://www.mathworks.com/).

### Demographic comparisons

In NC, MCI, and ADD groups, differences between men and women were assessed for demographic variables including age, YOE, ApoE status, handedness, and amyloid status. A chi-square test was used to examine categorical variables (*ε*2 and *ε*4 carrier status, amyloid status, and handedness) and a two-sample *t* test was used to determine differences among continuous variables (ages and YOE).

### Analysis of covariance (ANCOVA): sex specific cognition and brain structure changes

For all 824 subjects, to investigate sex-differences of CT measures in NC, MCI, and ADD stages, we applied the following ANCOVA model (Eq. [1]) to examine whether CT measures were associated with sex, disease diagnosis (DX), or the interaction between sex and DX, with age, YOE, handedness,*ε*2 carrier status, *ε*4 carrier status, and total intracranial volume (TIV) as covariates:

1 $$\mathrm{CT}\ \mathrm{measures}\sim 1+\mathrm{Sex}+\mathrm{DX}+\mathrm{Sex}\times \mathrm{DX}+\mathrm{Age}+\mathrm{YOE}+\mathrm{Handedness}+\varepsilon 2+\varepsilon 4+\mathrm{TIV}$$

Since we were specifically interested in the effects of sex, DX, and the interaction between sex and DX in each of the 68 cortical thickness measures, uncorrected *p* values were corrected for 68x3 comparisons using the false discovery rate (FDR) method. The same ANCOVA model without TIV as a covariate was applied to RAVLT-Immediate and RAVLT-Delayed scores to investigate if there were significant sex, DX, or interaction effects in memory scores. Every CT measure and memory score was first normalized to *z*-score in all 824 subjects before input to the ANCOVA model.

### Moderation analysis: sex-specific thickness-cognition associations

For regions demonstrating significant interaction effects of sex and DX in the ANCOVA analysis, we were further interested in whether sex would moderate the cognition-thickness relationships across all subjects from NC to ADD. To this end, a moderation regression analysis was performed on all subjects for RAVLT-immediate score, with each significant regional thickness measure in ANCOVA as the independent variable, sex as the moderator, and DX, age, YOE, handedness, *ε*2, and *ε*4 carrier status as covariates. Moderation regression analyses were followed by correlation analyses to evaluate cognition-thickness associations within men and women, respectively, partialling out effects of the same set of covariates. The same moderation analysis was repeated in Aβ+ and Aβ− subjects separately to further delineate the sex moderation effect on cognition-thickness relationships in subjects belonging to our different diagnostic groups.

### Sex classification in each diagnostic group using cortical thickness features

To further jointly evaluate multivariate sex differences in whole-brain CT measures from NC to ADD, 68 CT measures were used as features to classify men from women in NCAβ−, NCAβ+, MCIAβ−, MCIAβ+, and ADDAβ+ groups, respectively. Briefly, in each diagnostic group, a linear support vector machine (SVM) classifier was used to evaluate the classification performance with a leave-one-out cross-validation strategy. More specifically, in each diagnostic group, CT measures were first adjusted for covariate effects of age, YOE, handedness, *ε*2, and *ε*4 carrier status, and a linear SVM classifier was then trained on N-1 subjects with adjusted CT measures as features and then tested on the remaining 1 subject. In this strategy, every subject was being left out as the testing subject once, and the inverse probability weighting was applied to offset sex-imbalances. The test results for every subject were finally compared with the true sex labels. Sensitivity, specificity, accuracy, and area under the receiver operating characteristic (ROC) curves were used to evaluate the classifier performance.

## Results

### Demographics

Demographics and sex differences of demographic variables in NC, MCI, and ADD subjects are summarized in Table 1.

**Table 1:**
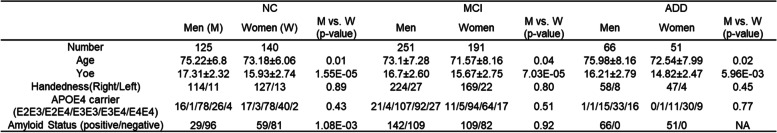
Subjects’ demographics of 824 subjects

Handedness and ApoE genotypes are matched between men and women in NC, MCI, and ADD subjects (Table 1). Overall, men are older than women in NC (*p*=0.01), and MCI (*p*=0.04) and ADD (*p*=0.02) subjects, with an average age difference of 2.04 years in NC subjects, 1.53 years in MCI subjects and 3.44 years in ADD subjects. Furthermore, men are more highly educated than women, as years of education are significantly higher in men than women in NC (*p*<0.001), MCI (*p*<0.001), and ADD (*p*=0.006) subjects.

*ANCOVA analysis: sex specific cognition and brain structure changes along diagnostic groups*

### Cognition

**Fig. 1:**
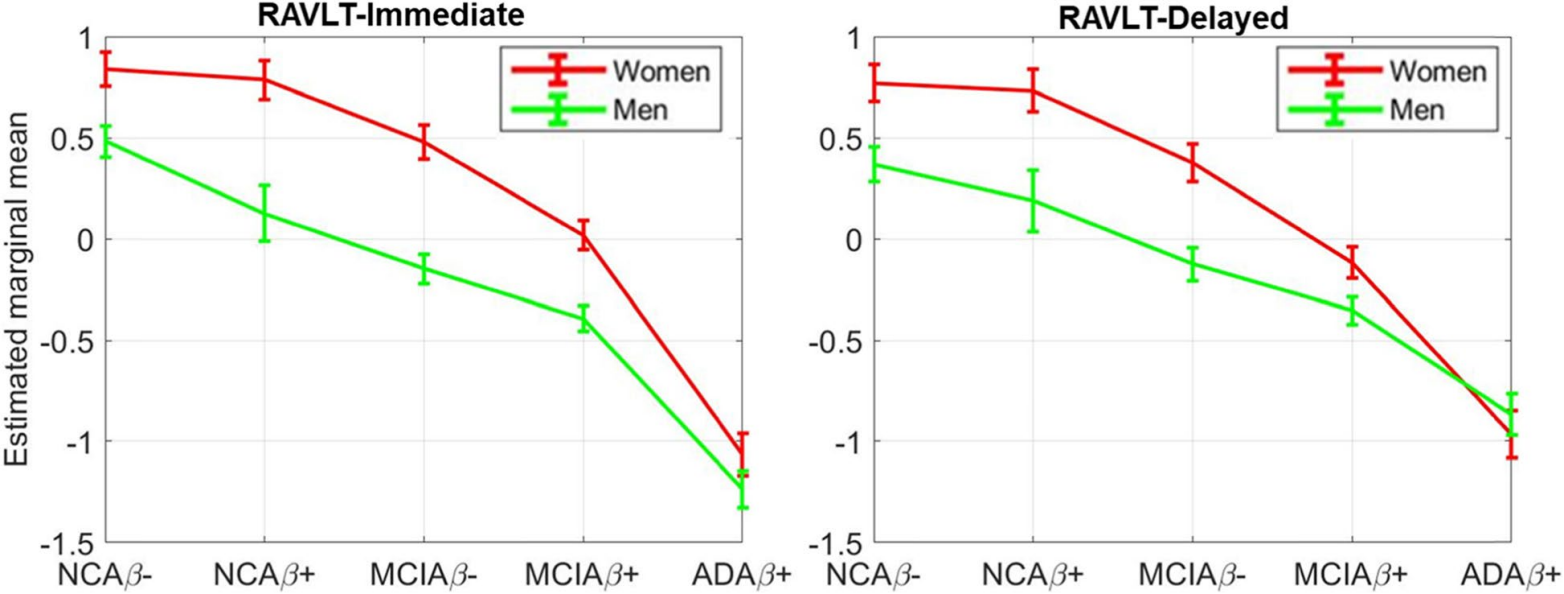
Memory scores: sex-specific changing trajectories of RAVLT scores across stages. Significant sex (*p*<0.001) and DX (*p*<0.001) effects are observed for RAVLT-Immediate *(Left)* and RAVLT-Delayed scores (*right*), respectively. Statistically significant interaction effect is observed for RAVLT-Delayed score (*p*=0.01, *Right*) and trend-level interaction effect is found for RAVLT-immediate score (*p*=0.058, *left*). Estimated marginal means of the interaction effect in ANCOVA are plotted for women (red) and men (green)

Figure 1 plots the sex-specific changing trajectories of RAVLT-Immediate (left) and RAVLT-Delayed (right) scores along individuals of our diagnostic groups. Marginal means of the interaction effect in the ANCOVA model are plotted. Significant sex (*p*<0.001 and *p*<0.001) and DX (*p*<0.001 and *p*<0.001) effects in ANCOVA model are observed for RAVLT-Immediate and RAVLT-Delayed scores, respectively. In addition, a statistically significant interaction effect is observed for RAVLT-Delayed score (*p*=0.01) and a trend-level interaction effect is found for RAVLT-immediate score (*p*=0.058). Overall, women have significantly higher scores than men; sex-differences in both scores are evident in NCAβ−, NCAβ+, MCIAβ−, and MCIAβ+ groups, but these differences diminish in ADDAβ+ group.

### Structural brain measures: cortical thickness

**Fig. 2:**
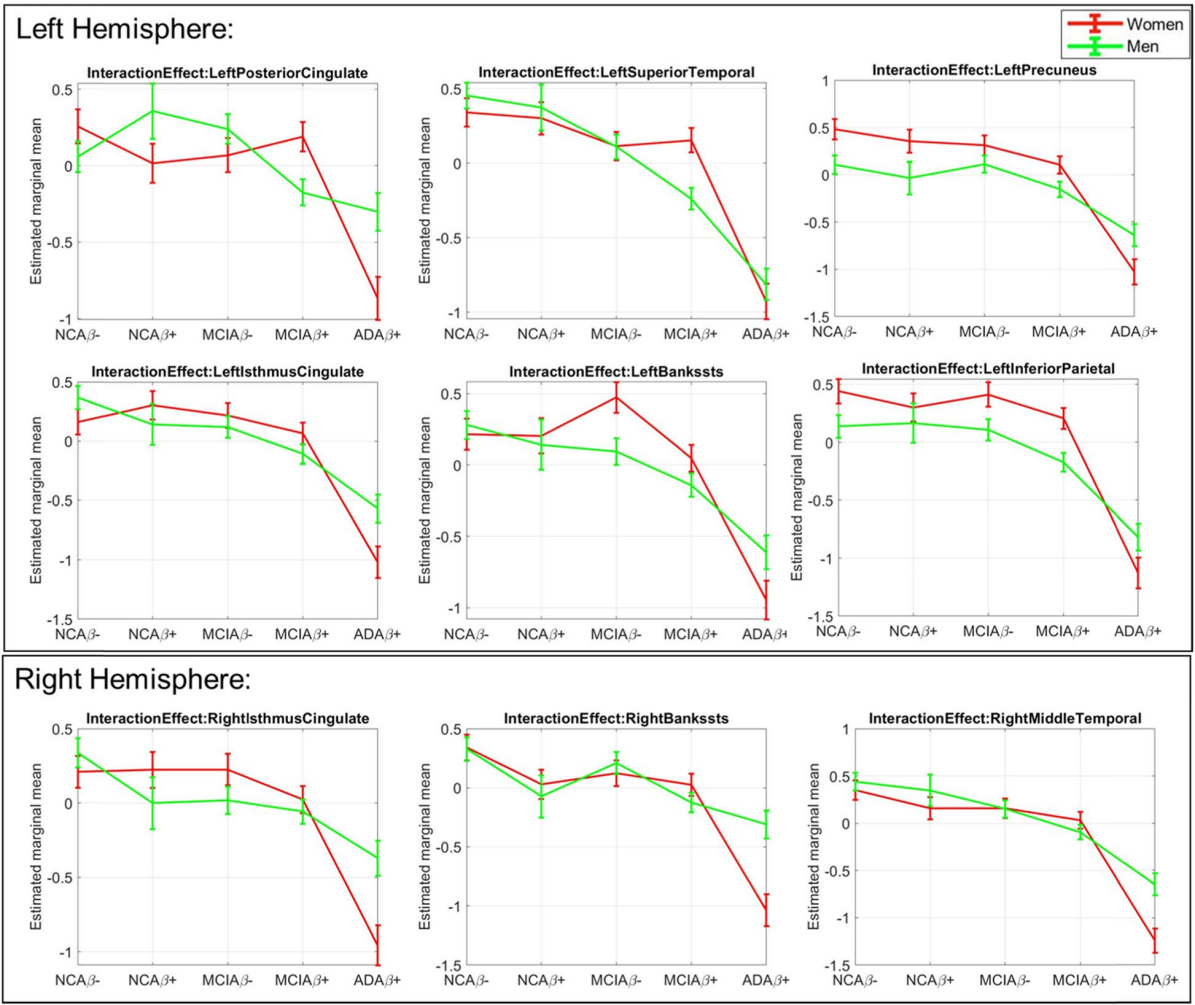
Cortical thickness measures: sex-specific changing trajectories of cortical thickness measures with significant interaction effects (FDR corrected *p*<0.05) between sex and diagnosis along NC, MCI, and AD stages in ANCOVA. Estimated marginal means of the interaction effect in ANCOVA are plotted for women (red) and men (green), respectively

Table 2 summarizes the sex, DX, and interaction effects in the ANCOVA model for all 68 CT measures in our sample, with uncorrected *p* values<0.05 listed and significant *p* values after false discovery rate (FDR) correction (*p*_corr_<0.05) highlighted in bold. Specifically, out of 68 brain regions, 55 regions demonstrate significant DX effects (*p*_corr_<0.05), and 14 regions demonstrate significant sex effects (*p*_corr_<0.05) in CT measures. For DX effect, significant declines across our diagnostic stages are evident in all regional CT measures, whereas for sex effects, women demonstrate greater CT measures in all 14 regions. More importantly, significant (*p*_corr_<0.05) interaction effects of CT measures are found in 9 brain regions, including bilateral cingulate cortex, bilateral temporal regions, and left parietal regions including precuneus and inferior parietal cortex (4^th^ and 8^th^ columns in Table 2). Figure 2 plots the different trajectories between men and women of these 9 regional thickness measures along the ADD stages. Marginal means of the interaction effect in the ANCOVA model are plotted for each thickness feature.

**Table 2:**
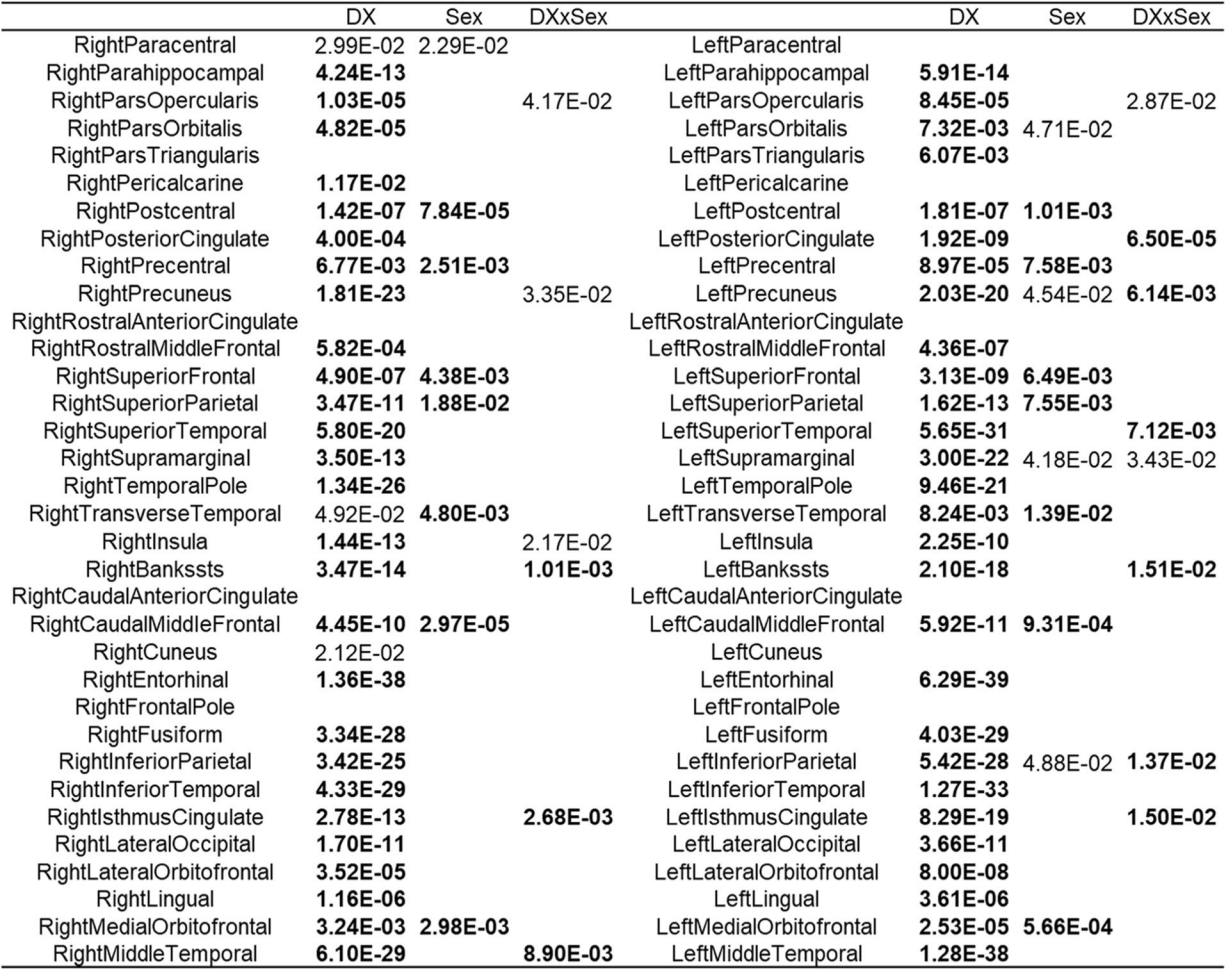
Cortical thickness measures ANCOVA results: *p* values of significant diagnosis (DX), sex, and interaction effects (uncorrected *p*<0.05) of cortical thickness measures in ANCOVA. Significant effects after FDR corrections are highlighted in bold. Out of 68 brain regions, 55 show significant DX effects, 14 show significant sex effects, and 9 show significant interaction effects in cortical thickness measures after FDR correction

### Sex moderates cognition-thickness associations across diagnosis

**Fig. 3:**
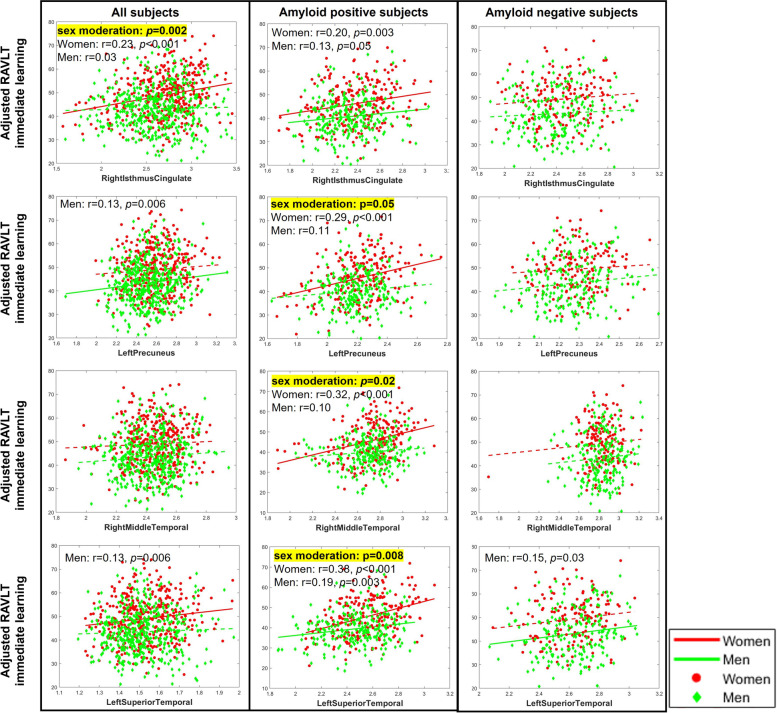
Relationships of verbal learning with regional thickness measures by sex in all subjects (left), amyloid positive subjects only (middle), and amyloid negative subjects only (right). Out of the 9 regions showing significant sex-dependent changing trajectories in ANCOVA, 4 regions are also showing significant sex moderation effects on associations between RAVLT-immediate learning score and regional thickness measure in all, or amyloid positive subjects and are plotting here for women (red) and men (green), separately. *P* values for significant sex-moderation effects are listed in the insets. Significant (*p*≤0.05) post hoc partial correlations (*r*) in women or men are also listed in the insets and represented by solid lines

Table 3 (A, left) summarizes the moderation analyses results for the 9 regions with significant interaction effects in ANCOVA. Significant sex moderation effects are observed between RAVLT-immediate scores and CT measures of right isthmus-cingulate (*p*=0.002) for all subjects across DX. As detailed in Fig. 3 (left) and Table 3 (A, right), in all subjects, partial correlation analyses reveal that increased CT of right isthmus-cingulate is associated with better verbal learning in women (Pearson’s correlation (*r*) = 0.23, *p*<0.001), but not in men (*r* = 0.03). When we stratify subjects based on Aβ status, we found that this significant cognition-thickness association is driven by Aβ+ subjects (i.e., subjects along the *ADD continuum*), with partial correlation analyses again showing significant positive correlations between these two measures in women only (Fig. 3 (center) and Table 3 (B, right)). These cognition-thickness relationships are not observed for Aβ− subjects (Fig. 3 (right) and Table 3 (C, right)).

**Table 3:**
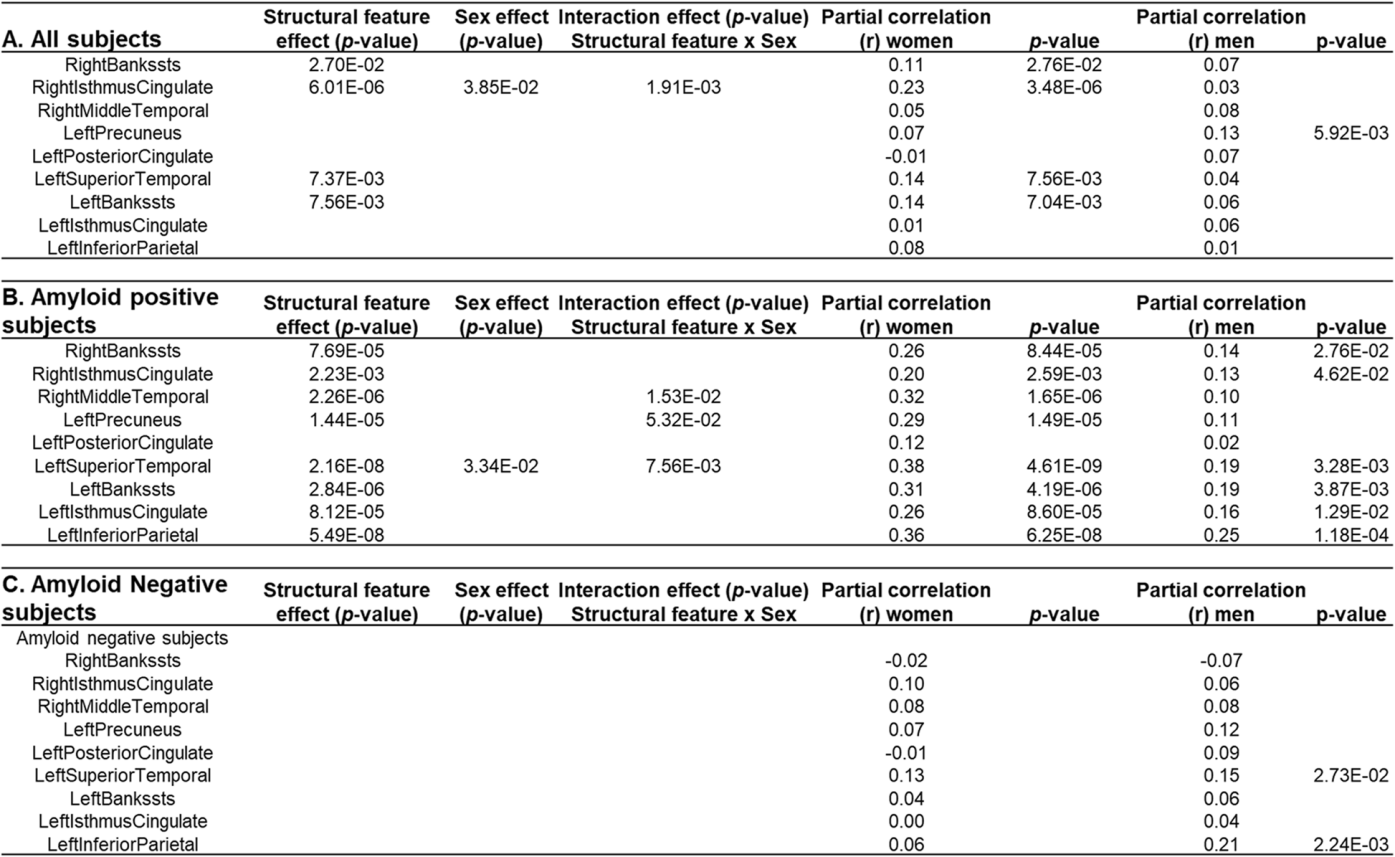
Sex moderate relationships of cognition (RAVLT immediate learning score) with brain cortical thickness measures in all subjects (A), amyloid positive subjects (B), and amyloid negative subjects (C). Nine regions with significant interaction effect in ANCOVA analyses are selected. Significant *p* values (*p*≤0.05) of the moderation analyses (left) and post hoc partial correlation (r) analyses (right) are listed

In Aβ+ subjects only, additional significant sex-moderation effects are found in right middle-temporal (*p*=0.02), left precuneus (*p*=0.05), and left superior temporal regions (*p*=0.008). A similar pattern of significantly stronger positive cognition-thickness associations in women than men were observed for all three regions in these subjects (Fig. 3 (center)), as revealed by the partial correlation values listed in Table 3 (B, right).

### Sex classification results using machine learning technique

**Fig. 4:**
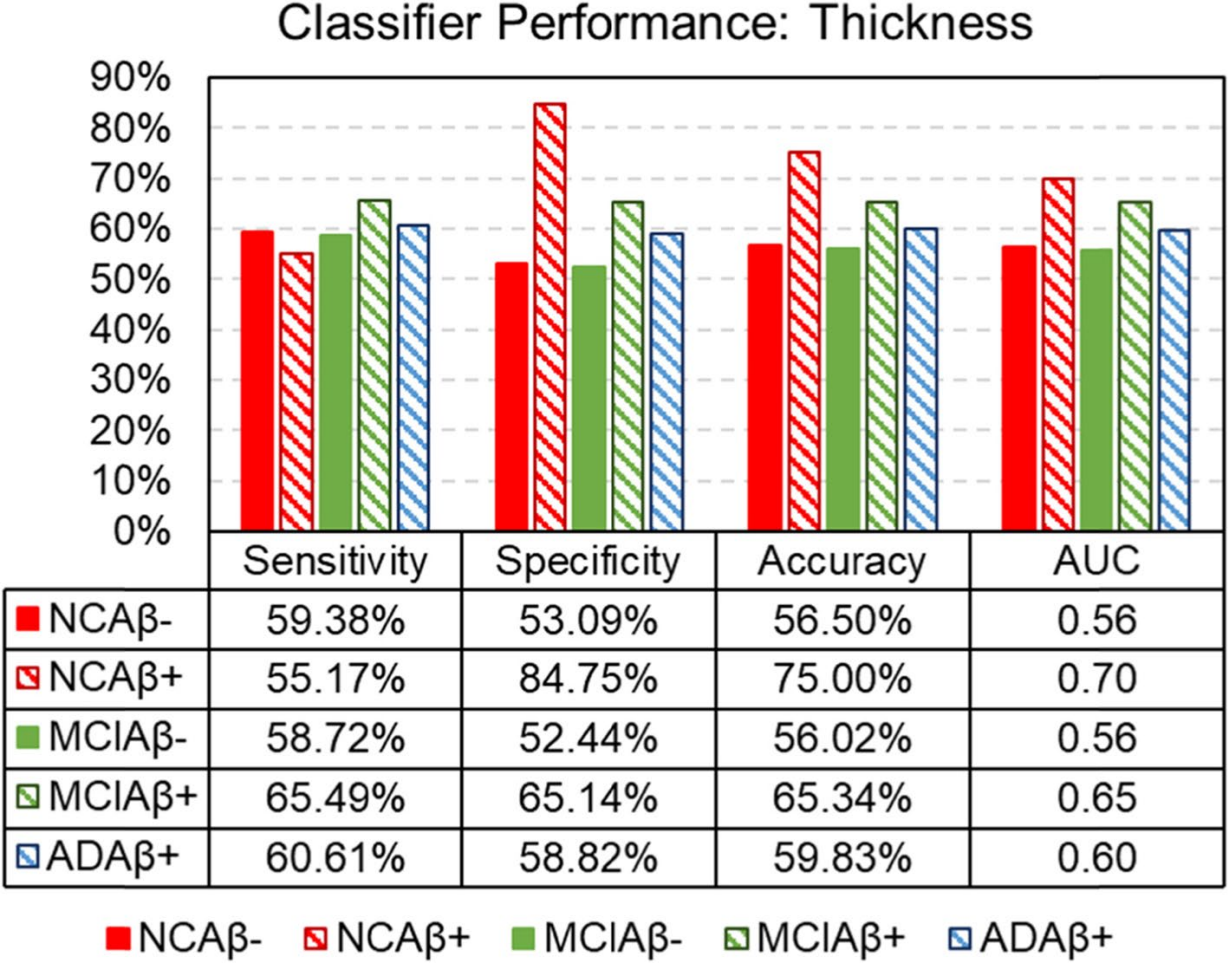
Support vector machine performance of sex classification in each diagnostic group with 68 cortical thickness measures as input features using 824 subjects. Sensitivity, specificity, classification accuracy, and area under the ROC curves are shown. Abbreviations: AUC area under the ROC curves

Figure 4 shows the sensitivity, specificity, accuracy, and area under the ROC curves of classification between men and women using CT measures as features in each diagnostic group. In NC subjects, CT measures can classify men from women at an accuracy of 75.00% and 56.50% for NCAβ+ and NCAβ− subjects, respectively. These accuracies drop to 65.34% and 56.02% for MCIAβ+ and MCIAβ− subjects and to 59.83% for ADDAβ+ subjects. To determine the chance levels of these accuracies, we performed 1000-run permutation tests (supplementary S2). The 95th percentiles of the permutation accuracies are 56.44%, 59.72%, 56.31%, 55.47%, and 57.76% in NCAβ−, NCAβ+, MCIAβ−, MCIAβ+, and ADDAβ+, respectively. Thus, the observed classification accuracies in NCAβ+, MCIAβ+, and ADAβ+ are statistically significant at *p*<0.05 level relative to these chance levels.

## Discussion

As hypothesized, this study showed sex differences in CT and memory performance in NC, MCI, and ADD individuals. Women showed greater CT in several AD-relevant brain regions as well as more stable CT and memory performances, compared to men, from NC to MCI. However, women showed greater cross-sectional reduction in CT and memory from MCI to ADD. Where CT differed by sex, women, but not men, evidenced an association between greater CT in selected regions and better verbal learning, and this finding was particularly notable when analyses were limited to Aβ+ individuals (i.e., those on the AD continuum).

Regions where women showed different CT trajectories, compared to men, included the precuneus, the inferior parietal cortex, and isthmus-cingulate, located at the posterior end of the cingulate cortex, confirming the results from Sangha et al. (2021) [17] obtained in a larger sample (ADNI and AIBL datasets), albeit in that study the authors did not explore the cognitive relationships.

The precuneus is a complex area involved in recollection and episodic memory retrieval [25] and one of the first regions to be affected by Aβ deposition [26], an important observation since the post hoc plots within the ADD continuum groups (NCAβ+, MCIAβ+, and ADD subjects only) showed similar trajectories as plots for all NC, MCI, and ADD individuals (Supplement S3). This area lies posterior and superior to the posterior cingulate cortex. The cingulate cortex plays a fundamental role in many cognitive, motor, and emotional functions [27], and its posterior part terminates at the isthmus of the cingulate gyrus. Although the specific function of the isthmus-cingulate cortex is not well understood, there is evidence of its involvement in episodic memory [28], other cognitive functions, and cortical anatomy changes by ADD pathology [29].

Precuneus, isthmus-cingulate and the inferior parietal cortex are all part of the posterior Default Mode Network (DMN), which supports autobiographical memory, future planning, records of bodily sensations, self-reported mental processes, and monitoring psychological states [30–32]. This network seems to play a key role in the vulnerability to ADD pathology [30, 33]. Our results indicate potential sex-dependent DMN-region differences along the ADD stages, with women showing a pattern of maintenance of CT from NC to MCI and steeper loss from MCI to ADD.

As with CT measures, women showed significantly higher learning and memory scores than men, with more stability from NC to MCI and greater cross-sectional decline from MCI to ADD. Our results are consistent with women showing ADD-relevant memory and brain-based reserve, which impact early trajectory when in place and later trajectory when lost. This pattern (i.e., early resilience, followed by steeper decline) is consistent with that found in studies of individuals with higher cognitive reserve based on education levels [34–36]. At the same time, education does not explain the current effects, and in fact, women in our sample have lower education than men, yet still evidence a reserve-like pattern. This provides further support for the idea of domain-specific cognitive reserve, specifically verbal memory reserve, in women.

The cross-sectional characteristics of potential CT-based brain reserve mirror the pattern seen in memory findings, and women but not men show a link between greater CT and better memory. Our CT reserve effect seems to act at the stage of NC and MCI, whereas during the advanced stage of neurodegeneration (ADD), women’s brain, and cognitive decline is greater compared to men. Overall, these findings lend support to the idea that regional CT maintenance may play a role in early memory resilience in women and are consistent with recent structural [17] and functional findings [5].

CT can classify men from women with descending accuracies in NC, MCI, and ADD individuals and that this approach is most successful in NCAβ+ individuals. This finding supports the hypothesis that early structural brain differences may contribute to different early trajectories of decline in men and women with ADD. It suggests that CT sex differences may be less relevant in normal aging as compared to the MCI and ADD, as well as less relevant with progression to ADD. This finding indicates that it may be important to examine differences between Aβ+ and Aβ− women and to consider these differences when designing early interventions or clinical trials for women.

### Limitation

Our study has limitations, for example our interpretation of reserve does not consider other contributors, such as occupational status, or other physical and cognitively stimulating activities, and does not speak to the reasons that women may gain memory or related brain reserve. In addition, the machine learning approach is unable to explore individual differences that may play a role in ADD pathogenesis. Finally, the ADNI sample is highly educated and predominantly White, and work in other samples will be important to ensure generalizability. On the other hand, our study has strengths in our consideration of multimodal sex differences in a relatively large sample and with consideration of the presence of brain amyloid.

## Conclusion

In conclusion, we found that women show more stable memory and CT than men from NC to MCI, and steeper thickness declines from MCI to ADD in regions including the precuneus, temporal lobe, and cingulate gyrus, areas that play a key role in memory and are among the most affected by the ADD neuropathology. Using CT as structural measure, our machine learning approach was able to classify men from women with good accuracy, especially in NC Aβ+ subjects, losing accuracy with progressive cognitive impairment. Future structural and functional MRI studies should consider sex as a factor of interest rather than a covariate and should consider domain-specific cognitive reserve and early CT-based brain reserve in women in ADD.

## Supplementary Information

**Additional file 1: Supplementary S1: Table S1.** 68 cortical regions of interest from Desikan-Killiany atlas. **Supplementary S2.** Computation of chance levels for classification between men and women. To determine the chance levels for sex classification accuracies, we performed 1000-run permutation tests in NCAβ-, NCAβ+, MCIAβ-, MCIAβ+ and ADAβ+ subjects, respectively. For each permutation run, we randomly shuffled the sex labels in that diagnostic group and followed the exact original classification schema described in section 2.2.4. Accuracy of each permutation run was finally recorded for each diagnostic group. **Figure S2.** below plots the empirical distribution of the classification accuracies in NCAβ-, NCAβ+, MCIAβ-, MCIAβ+ and ADAβ+ subjects during 1000 permutation runs, respectively. The classification accuracy with true labels and the 95^th^ percentile of empirical accuracy distribution are plotted in solid red line and dashed red line, respectively. Therefore, the classification accuracies in NCAβ+, MCIAβ+ and AD Aβ+ subjects are statistically significant at *p*=0.05 level. **Figure S2.** Chance level for classification accuracies in NCAβ-, NCAβ+, MCIAβ-, MCIAβ+ and ADAβ+. **Supplement S3. Figure S3.** Post-hoc plots within subjects along the ADD continuum (NCAβ+, MCIAβ+ and ADD subjects) of cortical thickness measures with significant interaction effects (FDR corrected *p*<0.05) between sex and diagnosis in original ANCOVA (Fig. 2). Estimated marginal means of the interaction effect in ANCOVA are plotted for women (red) and men (green), respectively.

## Data Availability

ADNI subjects ID used in this article will be made available upon reasonable request.

## References

[1] Caldwell JZK, Berg JL, Cummings JL, Banks SJ (2017). Alzheimer’s Disease Neuroimaging Initiative. Moderating effects of sex on the impact of diagnosis and amyloid positivity on verbal memory and hippocampal volume. Alzheimers Res Ther..

[2] Caldwell JZK, Berg JL, Cummings JL, Banks SJ (2018). Sex moderates theimpact of diagnosis and amyloid PET positivity on hippocampal subfield volume. J Alzheimer’s Dis..

[3] Caldwell JZK, Cummings JL, Banks SJ (2019). Cognitively normal women with Alzheimer’s disease proteinopathy show relative preservation of memory but not of hippocampal volume. Alz Res Ther..

[4] Caldwell JZK, Zhuang X, Leavitt MJ, Banks SJ, Cummings J, Cordes D. Sex moderates amyloid and apolipoprotein ε4 effects on default mode network connectivity at rest. Frontiers in. Neurology. 2019b, 2019b:1–9. 10.3389/fneur.2019.00900. 10.3389/fneur.2019.00900 PMC6710397 31481928

[5] Cieri F, Yang Z, Cordes D, Jessica Caldwell ZK. Alzheimer’s Disease Neuroimaging Initiative. Sex differences of brain functional topography revealed in normal aging and Alzheimer’s disease cohort. J Alzheimers Dis. 2021a. 10.3233/JAD-201596 Epub ahead of print. PMID: 33612547. 10.3233/JAD-201596 PMC8793667 33612547

[6] Jack CR, Wiste HJ, Weigand SD, Knopman DS, Vemuri P, Mielke MM, Lowe V, Senjem ML, Gunter JL, Machulda MM, Gregg BE, Pankratz VS, Rocca WA, Petersen RC (2015). Age, sex, and APOEε4 effects on memory, brain structure, and β-amyloid across the adult life span. JAMA Neurol..

[7] Nebel RA, Aggarwal NT, Barnes LL (2018). 2018. Understanding the impact of sex and gender in Alzheimer’s disease: a call to action. Alzheimers Dement..

[8] Association A (2021). Alzheimer’s disease facts and figures. SPECIAL REPORT.

[9] Ardekani BA, Convit A, Bachman AH (2016). Analysis of the MIRIAD data shows sex differences in hippocampal atrophy progression. J Alzheimers Dis..

[10] Skup M, Zhu H, Wang Y (2011). Sex differences in grey matter atrophy patterns among AD and aMCI patients: results from ADNI. Neuroimage..

[11] Buckley RF, Mormino EC, Amariglio RE (2018). Sex, amyloid, and APOE ε4 and risk of cognitive decline in preclinical Alzheimer’s disease: findings from three well-characterized cohorts. Alzheimers Dement..

[12] Buckley RF, Mormino EC, Rabin JS (2019). Sex differences in the association of global amyloid and regional tau deposition measured by positron emission tomography in clinically normal older adults. JAMA Neurol..

[13] Brunet HE, Caldwell JZK, Brandt J, Miller JB (2020). Influence of sex differences in interpreting learning and memory within a clinical sample of older adults. Neuropsychol Dev Cogn B Aging Neuropsychol Cogn..

[14] Sundermann EE, Maki P, Biegon A (2019). Sex-specific norms for verbal memory tests may improve diagnostic accuracy of amnestic MCI. Neurology..

[15] Sundermann EE, Biegon A, Rubin LH, Lipton RB, Landau S, Maki (2017). Does the female advantage in verbal memory contribute to underestimating Alzheimer’s disease pathology in women versus men?. J Alzheimers Dis.

[16] Sundermann EE, Tran M, Maki PM, Bondi MW (2018). Sex differences in the association between apolipoprotein E e4 allele and Alzheimer’s disease markers. Alzheimers Dement (Amst)..

[17] Sangha O, Ma D, Popuri K, Stocks J, Wang L, Faisal Beg M. Alzheimer’s Disease Neuroimaging Initiative, Australian Imaging Biomarkers and Lifestyle flagship study of ageing. Structural volume and cortical thickness differences between males and females in cognitively normal, cognitively impaired and Alzheimer’s dementia population. Neurobiol Aging. 2021;11165, ISSN 0197-4580. 10.1016/j.neurobiolaging.2021.05.018. 10.1016/j.neurobiolaging.2021.05.018 34216846

[18] Seo SW, Im K, Lee JM, Kim ST, Ahn HJ, Go SM, Kim SH, Na DL (2011). Effects of demographic factors on cortical thickness in Alzheimer’s disease. Neurobiol Aging.

[19] Stern Y, Gurland B, Tatemichi TK, Tang MX, WilderD, and Mayeux R. (1994). Influence of education and occupation on the incidence of Alzheimer’s disease. J Am Med Assoc.

[20] Arenaza-Urquijo EM, Wirth M, Chételat G (2015). Cognitive reserve and lifestyle: moving towards preclinical Alzheimer’s disease. Front Aging Neurosci.

[21] Kotsiantis SB, Zaharakis ID, Pintelas PE (2006). Machine learning: a review of classification and combining techniques. Artif Intell Rev.

[22] Querbes O, Aubry F, Pariente J, Lotterie JA, Démonet JF, Duret V, Puel M, Berry I, Fort JC, Celsis P (2009). Alzheimer’s Disease Neuroimaging Initiative. Early diagnosis of Alzheimer’s disease using cortical thickness: impact of cognitive reserve. Brain.

[23] Sorensen L (2016). Early detection of Alzheimer’s disease using MRI hippocampal texture. Hum Brain Mapp.

[24] Desikan RS, Ségonne F, Fischl B, Quinn BT, Dickerson BC, Blacker D, Buckner RL, Dale AM, Maguire RP, Hyman BT, Albert MS (2006). An automated labeling system for subdividing the human cerebral cortex on MRI scans into gyral based regions of interest. Neuroimage.

[25] Borsook D, Maleki N, Burstein R. Chapter 42 - Migraine. In: Zigmond MJ, Rowland LP, Coyle JT, editors. Neurobiology of Brain Disorders: Academic Press; 2015. p. 693–708.

[26] Buckner RL, Sepulcre J, Talukdar T, Krienen FM, Liu H, Hedden T, Andrews-Hanna JR, Sperling RA, Johnson KA (2009). Cortical hubs revealed by intrinsic functional connectivity: mapping, assessment of stability, and relation to Alzheimer’s disease. J Neurosci..

[27] Cera N, Esposito R, Cieri F, Tartaro A (2019). Altered cingulate cortex functional connectivity in normal aging and mild cognitive impairment. Front Neurosci.

[28] Valenstein E, Bowers D, Verfaellie M, Heilman KM, Day A, Watson RT (1987). Retrosplenial amnesia. Brain..

[29] Yang H, Xu H, Li Q, Jin Y, Jiang W, Wang J, Wu Y, Li W, Yang C, Li X, Xiao S, Shi F, Wang T (2019). Study of brain morphology change in Alzheimer’s disease and amnestic mild cognitive impairment compared with normal controls. Gen Psychiatr..

[30] Cieri F, Esposito R (2018). Neuroaging through the lens of the resting state networks. Biomed Res Int..

[31] Cieri F, Esposito R (2019). Psychoanalysis and neuroscience: the bridge between mind and brain. Frontiers in Psychology..

[32] Cieri F, Zhuang X, Caldwell JZK, Cordes D (2021). Brain entropy during aging through a free energy principle approach. Front. Hum. Neurosci..

[33] Greicius MD, Krasnow B, Reiss AL, Menon V (2003). Functional connectivity in the resting brain: a network analysis of the default mode hypothesis. Proc Natl Acad Sci U S A..

[34] Stern Y, Arenaza-Urquijo EM, Bartrés-Faz D, Belleville S, Cantilon M, Chetelat G, Ewers M, Franzmeier N, Kempermann G, Kremen WS, Okonkwo O, Scarmeas N, Soldan A, Udeh-Momoh C, Valenzuela M, Vemuri P (2020). Vuoksimaa E; the Reserve, Resilience and protective factors PIA empirical definitions and conceptual frameworks workgroup. Whitepaper: Defining and investigating cognitive reserve, brain reserve, and brain maintenance. Alzheimers Dement..

[35] Stern Y, Albert S, Tang MX, Tsai WY (1999). Rate of memory decline in AD is related to education and occupation: cognitive reserve?. Neurology..

[36] Scarmeas N, Albert SM, Manly JJ, Stern Y (2006). Education and rates of cognitive decline in incident Alzheimer’s disease. J Neurol Neurosurg Psychiatry.

