# Supplementary material for: Relationship of sex differences in cortical thickness and memory among cognitively healthy subjects and individuals with mild cognitive impairment and Alzheimer disease
Source: Alzheimers Res Ther. 2022 Feb 22;14:36. doi: 10.1186/s13195-022-00973-1 (PMC8864917; doi:10.1186/s13195-022-00973-1)
Supplement: Supplementary file 1 — Additional file 1: Supplementary S1: Table S1. 68 cortical regions of interest from Desikan-Killiany atlas. Supplementary S2. Computation of chance levels for classification between men and women. To determine the chance levels for sex classification accuracies, we performed 1000-run permutation tests in NCAβ-, NCAβ+, MCIAβ-, MCIAβ+ and ADAβ+ subjects, respectively. For each permutation run, we randomly shuffled the sex labels in that diagnostic group and followed the exact original classification schema described in section 2.2.4. Accuracy of each permutation run was finally recorded for each diagnostic group. Figure S2. below plots the empirical distribution of the classification accuracies in NCAβ-, NCAβ+, MCIAβ-, MCIAβ+ and ADAβ+ subjects during 1000 permutation runs, respectively. The classification accuracy with true labels and the 95th percentile of empirical accuracy distribution are plotted in solid red line and dashed red line, respectively. Therefore, the classification accuracies in NCAβ+, MCIAβ+ and AD Aβ+ subjects are statistically significant at p=0.05 level. Figure S2. Chance level for classification accuracies in NCAβ-, NCAβ+, MCIAβ-, MCIAβ+ and ADAβ+. Supplement S3. Figure S3. Post-hoc plots within subjects along the ADD continuum (NCAβ+, MCIAβ+ and ADD subjects) of cortical thickness measures with significant interaction effects (FDR corrected p<0.05) between sex and diagnosis in original ANCOVA (Fig. 2). Estimated marginal means of the interaction effect in ANCOVA are plotted for women (red) and men (green), respectively. [file 13195_2022_973_MOESM1_ESM.docx]

## Supplementary S1.

Table S1. 68 cortical regions of interest from Desikan-Killiany atlas.


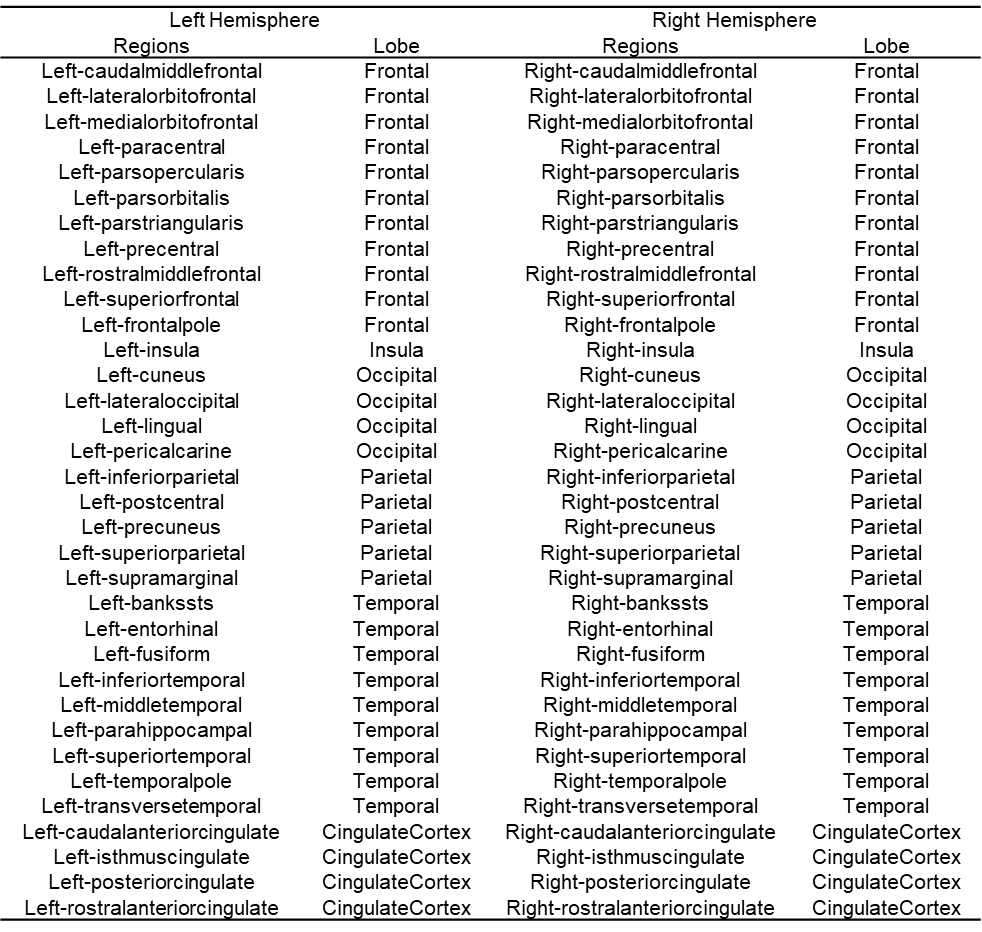


## Supplementary S2.

**Computation of chance levels for classification between men and women.**

To determine the chance levels for sex classification accuracies, we performed 1000-run permutation tests in NCAβ-, NCAβ+, MCIAβ-, MCIAβ+ and ADAβ+ subjects, respectively. For each permutation run, we randomly shuffled the sex labels in that diagnostic group and followed the exact original classification schema described in section 2.2.4. Accuracy of each permutation run was finally recorded for each diagnostic group.

Fig. S2 below plots the empirical distribution of the classification accuracies in NCAβ-, NCAβ+, MCIAβ-, MCIAβ+ and ADAβ+ subjects during 1000 permutation runs, respectively. The classification accuracy with true labels and the 95^th^ percentile of empirical accuracy distribution are plotted in solid red line and dashed red line, respectively. Therefore, the classification accuracies in NCAβ+, MCIAβ+ and AD Aβ+ subjects are statistically significant at *p*=0.05 level.


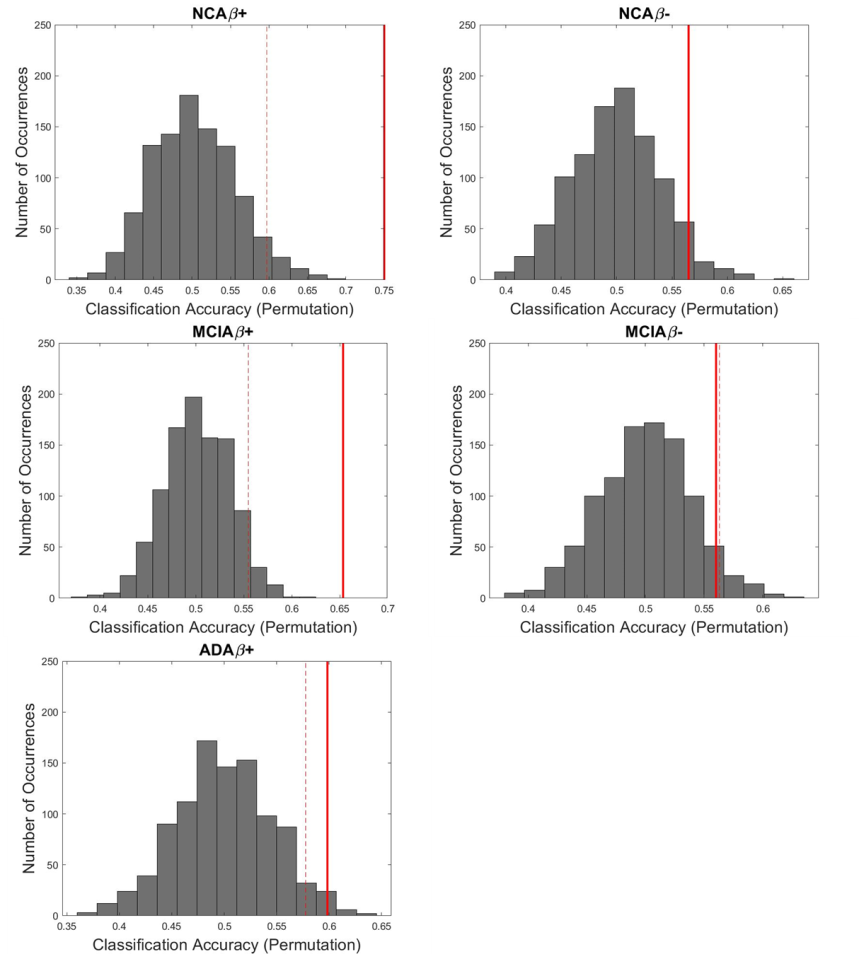


Fig. S2. Chance level for classification accuracies in NCAβ-, NCAβ+, MCIAβ-, MCIAβ+ and ADAβ+

## Supplement S3.


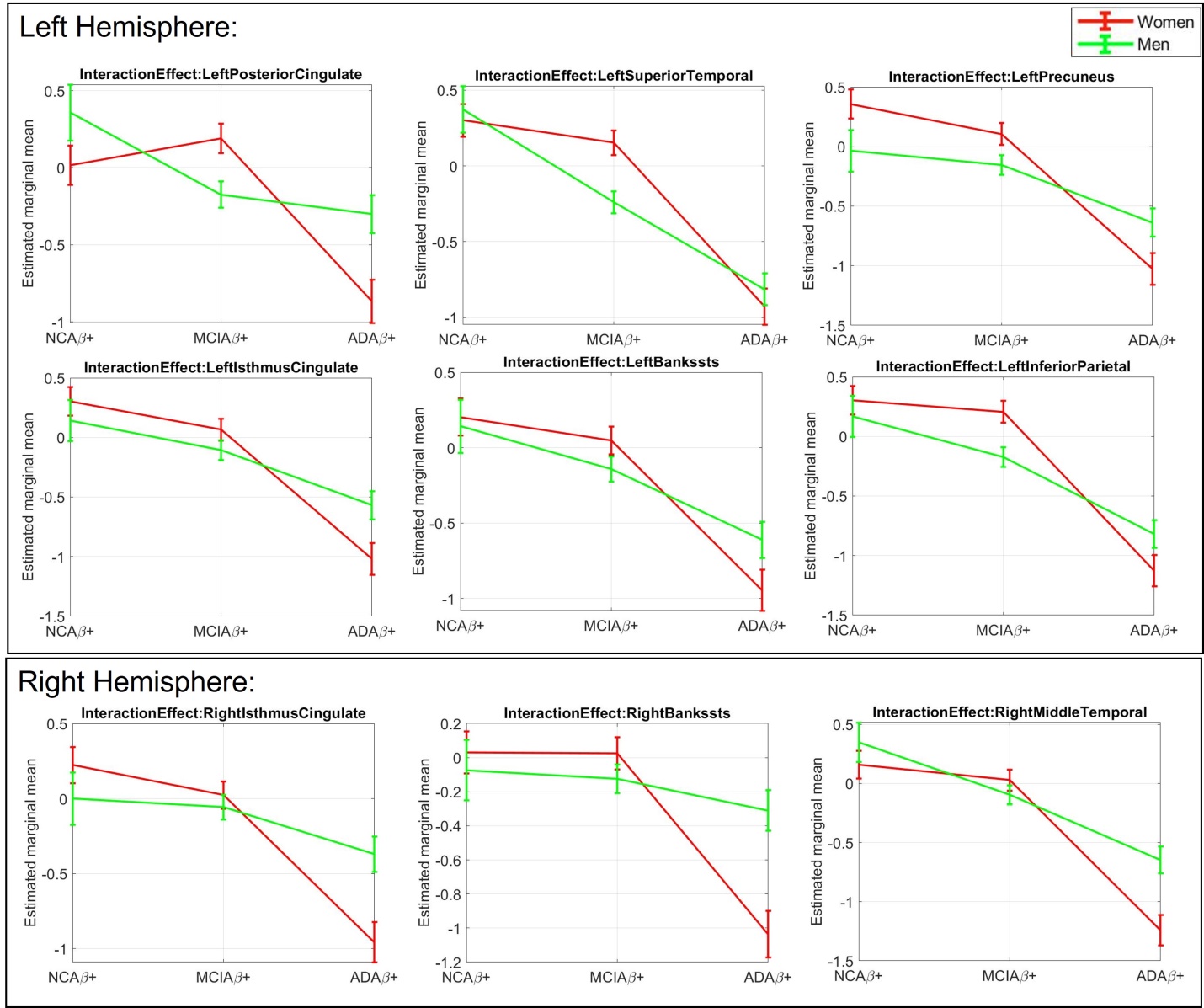


Fig. S3. Post-hoc plots within subjects along the ADD continuum (NCAβ+, MCIAβ+ and ADD subjects) of cortical thickness measures with significant interaction effects (FDR corrected *p*<0.05) between sex and diagnosis in original ANCOVA (Fig. 2). Estimated marginal means of the interaction effect in ANCOVA are plotted for women (red) and men (green), respectively.
